# Advances in Food Aroma Analysis: Extraction, Separation, and Quantification Techniques

**DOI:** 10.3390/foods14081302

**Published:** 2025-04-09

**Authors:** Dandan Pu, Zikang Xu, Baoguo Sun, Yanbo Wang, Jialiang Xu, Yuyu Zhang

**Affiliations:** 1 Food Laboratory of Zhongyuan, Beijing Technology and Business University, Beijing 100048, China; 18518351472@163.com (D.P.); 13526673386@163.com (Z.X.); 2 Key Laboratory of Geriatric Nutrition and Health Ministry of Education, Beijing Technology and Business University, Beijing 100048, China; sunbg@btbu.edu.cn (B.S.); wyb1225@163.com (Y.W.); xujialiang@btbu.edu.cn (J.X.); 3 Key Laboratory of Flavor Science of China General Chamber of Commerce, Beijing Technology and Business University, Beijing 100048, China

**Keywords:** aroma analysis, aroma extraction and isolation, quantification, artificial intelligence, flavoromics

## Abstract

Decoding the aroma composition plays a key role in designing and producing foods that consumers prefer. Due to the complex matrix and diverse aroma compounds of foods, isolation and quantitative analytical methods were systematically reviewed. Selecting suitable and complementary aroma extraction methods based on their characteristics can provide more complete aroma composition information. Multiple mass spectrometry detectors (MS, MS/MS, TOF-MS, IMS) and specialized detectors, including flame ionization detector (FID), electron capture detector (ECD), nitrogen–phosphorus detector (NPD), and flame photometric detector (FPD), are the most important qualitative technologies in aroma identification and quantification. Furthermore, the real-time monitoring of aroma release and perception is an important developing trend in the aroma perception of future food. A combination of artificial intelligence for chromatographic analysis and characteristic databases could significantly improve the qualitative analysis efficiency and accuracy of aroma analysis. External standard method and stable isotope dilution analysis were the most popular quantification methods among the four quantification methods. The combination with flavoromics enables the decoding of aroma profile contributions and the identification of characteristic marker aroma compounds. Aroma analysis has a wide range of applications in the fields of raw materials selection, food processing monitoring, and products quality control.

## 1. Introduction

Aroma is an important factor that determines the sensory quality of food, which is also the key driving force for consumers’ acceptance. Furthermore, aroma modification and improvement are useful and effective methods for food flavor iteration. Therefore, characterization of food aroma is a prerequisite for improving the aroma quality of food. Up to now, over 10,000 aroma compounds—including acids, alcohols, aldehydes, ethers, amines, phenols, esters, ketones, olefins, nitrogen-containing, sulfur-containing, and heterocyclic compounds, among others—have been identified among more than 270 kinds of foods [1,2]. Based on previous studies, aroma compounds in foods are derived mainly from three chemical processes, namely (1) enzymatic reactions, (2) microbial fermentation, (3) and chemical reactions [3,4,5,6]. Due to variance in formation pathways, the composition and concentration of aroma compounds can be affected by various factors, such as the raw materials, process methods, physical states, food matrixes, storage time, storage environment, etc. Therefore, the characterization and quantification of the characteristic aroma compounds of food are key to improving their sensory quality.

Currently, food aroma analysis can be divided into two parts. These are (1) the pretreatment of aroma separation and enrichment and (2) an instrumental qualitative and quantitative analysis of aroma. Aroma separation and enrichment are mainly conducted through solvent extraction using adsorbent materials and other equipment. Subsequently, gas chromatography combined with mass spectrometry technology, ion mobility spectrometry, and olfactory analysis are the main methods used to qualitatively analyze aroma compounds and elucidate their contribution to the overall aroma profile. Due to abundant aroma composition and low concentration, as well as complex matrix effects, selecting an appropriate aroma extraction method and effective analysis strategies are key to accurately characterizing food aroma and identifying the primary odorants.

Aroma is a determining factor in the sensory quality of food. It not only directly affects consumers’ acceptance but also plays an important role in food flavor iteration. Therefore, an accurate characterization of food aroma is of great significance for improving food sensory quality. This review aims to systematically introduce the latest trends in food aroma analysis methods, including aroma extraction, separation, identification, and quantification methods, as well as big data analysis (flavoromics) and artificial intelligence algorithms. Through an in-depth discussion of different methods, this study provides a comprehensive reference for relevant research and practical applications, promoting the development of the field of food aroma analysis.

## 2. Developing Trends in Aroma Analysis

### 2.1. Trends in Aroma Analysis Literature

**Figure 1:**
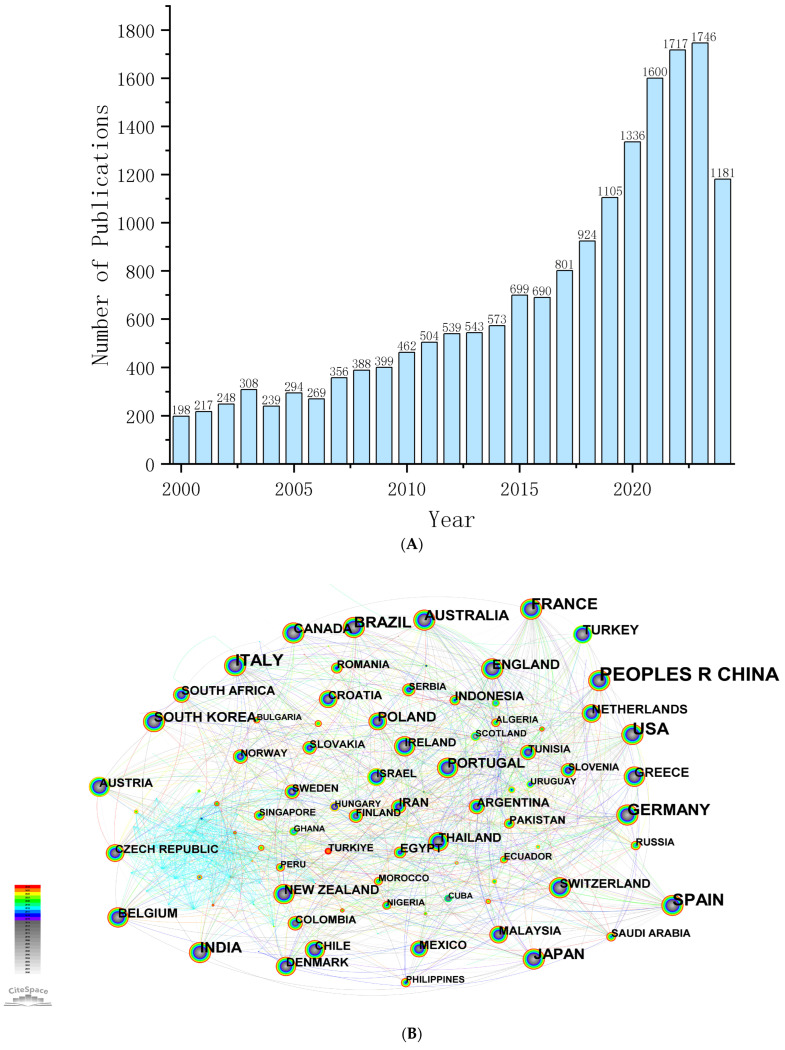

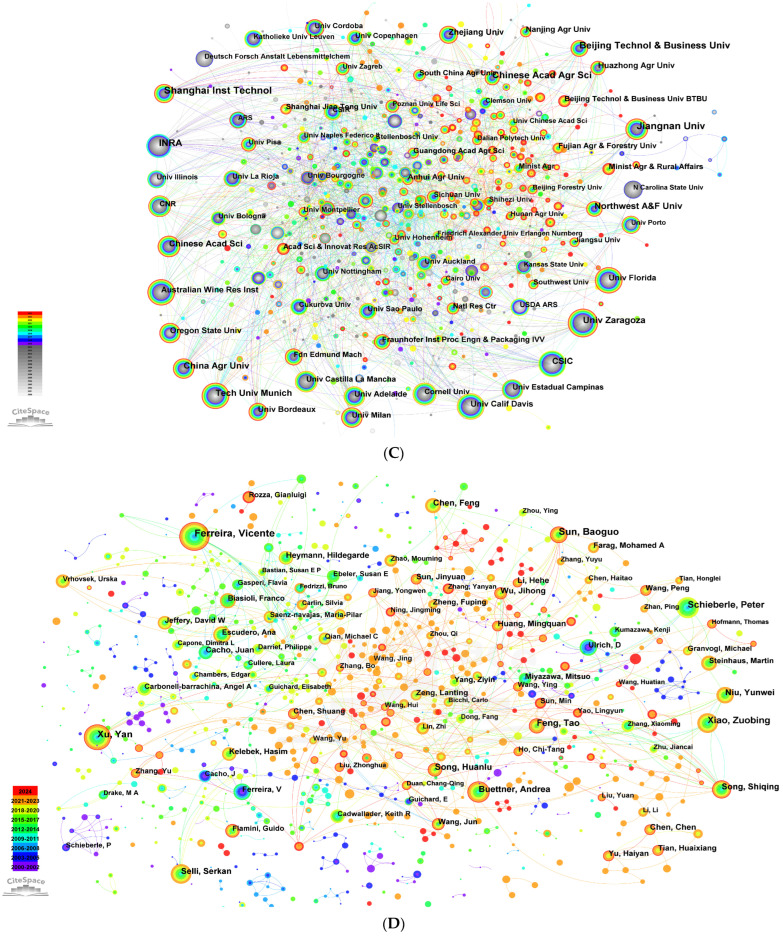

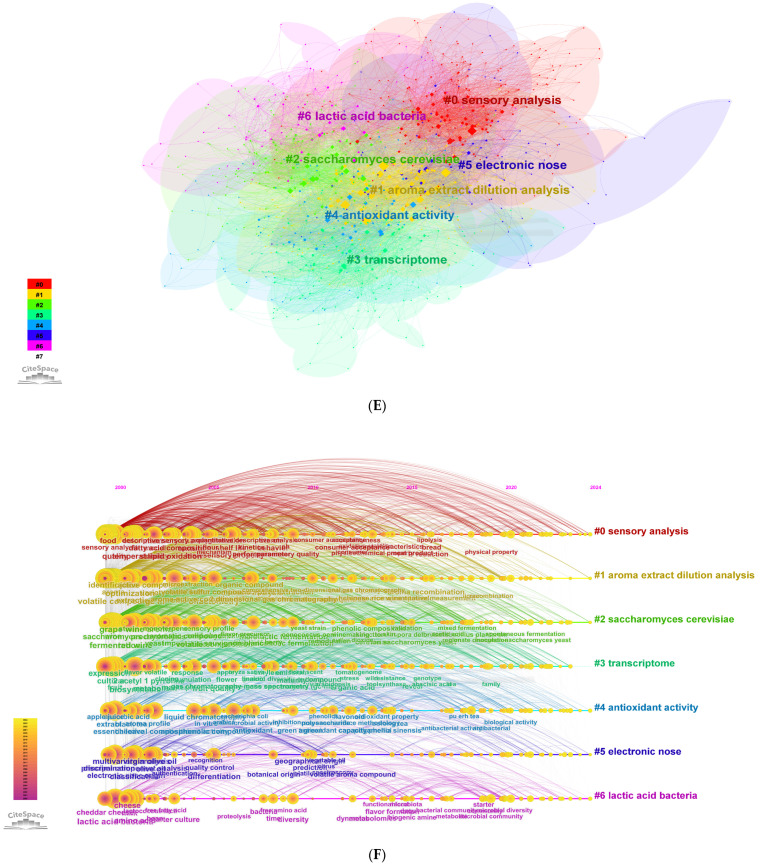
Current development trends of aroma analysis conducted by Citespace software 6.4.R1. (**A**) Number of publications from Web of Science data; (**B**) visualization of countries’ co-citation network; (**C**) visualization of institutional co-citation network; (**D**), visualization of author co-citation network; (**E**) network visualization map of keywords; (**F**) time zone view of keywords cluster.

In recent years, food flavor analysis investigation has been flourishing and developing due to the constant innovation in analytical techniques. The research trends during the last 24 years are summarized in Figure 1 based on the data collected from Web of Science (Web of Science Core Collection, January 2000 to August 2024. Aroma analysis was set as the keyword) by Citespace software 6.4.R1 [7]. Figure 1A shows that the number of publications in the aroma analysis field slowly increased each year from 2000 to 2016, and then exploded from 2016 to 2024, maintaining an annual growth trend. However, the literature was developed slowly in the early stage and gradually increased in the middle stage. Currently, the number of publications per year is still a gradual upward trend, indicating that the aroma analysis has great prospects and vitality.

A visualization of the international co-citation network (Figure 1B) shows that the collinear network consists of 149 nodes and 1165 connections, with a network density of 0.11, indicating that a total of 149 countries have conducted varying degrees of research in aroma analysis. Intermediary centrality (≥0.1) depends on the number of publications and the influence of the literature, which is an important indicator of the degree of influence exerted in a collinear network. The higher the intermediary centrality of a node, the greater its propagation influence. Of note, China, the United States, Spain, Italy, Germany, and France have published 4669, 1710, 1560, 1432, 1038, and 963 articles, respectively, ranking in the top six. Although China has the highest number of publications but with lower intermediary centrality (0.04), indicating that the international influence of China still needs to be strengthened. Importantly, the United States (0.21), France (0.19), and Germany (0.17) showed a significant international influence in aroma analysis. According to the statistical analysis (Figure 1C), INRA (268) had the most publications in France, the University of California, Davis (133) had the most in United States, the Shanghai Institute of Technology (380) had the most in China, and Technische Universität München (194) had the most in Germany. In addition, Vicente Ferreira (126), Baoguo Sun (113), Yan Xu (101), Zuobing Xiao (88), Peter Schieberle (86), Feng Tao (72) and Andrea Buettner (69) were the most published authors in aroma analysis worldwide (Figure 1D). The journals with the highest publication amount (over 1000 per year) on aroma analysis were Food Chemistry and Journal of Agricultural and Food Chemistry, followed by Food Research International, Foods, and LWT-Food Science and Technology with over 500 publications per year.

### 2.2. Evolutionary Patterns of Aroma Analysis

Clustering research hotspots in aroma analysis could help us keep up with cutting-edge development trends. Cluster results (Figure 1E; Q-value, 0.31; S-value, 0.66) showed that seven clusters, including # 0 sensory analysis, # 1 aroma extraction dilution analysis, # 2 *Saccharomyces cerevisiae*, # 3 transcriptome, # 4 antioxidant activity, # 5 electronic nose, and # 6 lactic acid bacteria, were assessed.

The time zone view of keyword clusters (Figure 1F) shows the process of development and correlations between different clusters. The #1 aroma extract dilution analysis (AEDA) has the highest citation count (2988) and a high outbreak frequency of 46.84, representing a milestone in aroma analysis [8]. Based on the AEDA, aroma-active compounds can be screened from many aroma compounds. Together, the AEDA, odor activity analysis, recombination, and omission/addition tests form molecular sensory science methods [9].

The #2 saccharomyces cerevisiae cluster has a high citation count (858) and is related to brewing and microbial fermentation. Saccharomyces cerevisiae is widely used in making various foods and drinks and is crucial for producing alcoholics, esters, and furanones [10,11,12].

The #3 transcriptome cluster, part of food omics, is related to the fermentation and maturation of living things. It can uncover aroma-related metabolic pathways and regulation mechanisms [13,14]. The #5 electronic nose, a rapid aroma analysis method, can create recognition models for food quality identification and control [15]. The microbial community and functional connectivity clusters are new research areas, suggesting a future trend towards the simultaneous development of flavor and health.

The software CiteSpace can evaluate the most frequently cited keywords (burst words) and their significance periods. It was used to select 22 keywords with the strongest burst intensity in aroma analysis research (Table 1). These keywords cover analytical methods, important aroma compounds, wine aroma, and microbial community-related aspects. Solid-phase microextraction (SPME) has the highest burst strength (90.83) from 2000 to 2011. Since its proposal in 1989, it has become an important aroma analysis tool due to its advantages. Correlation analysis and microbial community are recent keywords related to statistical analysis and fermented food aroma formation.

This study uses CiteSpace to analyze data from the Web of Science database from January 2000 to August 2024, taking “aroma analysis” as the keyword. It shows the development trends of the aroma analysis field for aspects such as the number of publications, cooperation among countries and institutions, and research hotspot clustering, providing context and a reference for the direction of future research.

## 3. Aroma Analytical Methods

### 3.1. Isolation of Aroma Compounds

**Figure 2:**
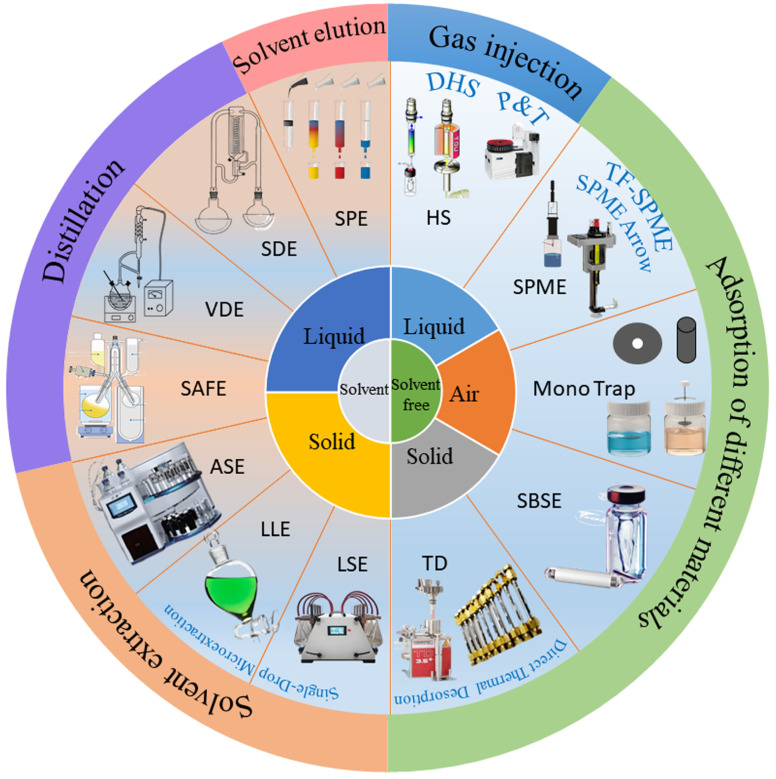
A summary of solvent and solvent-free aroma extraction methods and their corresponding food status (SPME, solid-phase microextraction; SBSE, stir-bar sorptive extraction; HS, headspace extraction; TD, thermal desorption; LLE, liquid–liquid extraction; LSE, liquid–solid extraction; SPE, solid-phase extraction; SAFE, solvent-assisted flavor evaporation; SDE, simultaneous distillation extraction; VDE, vacuum distillation extraction; ASE, accelerated solvent extraction).

Aroma extraction is crucial for accurate qualitative and quantitative analyses. However, some challenges occur during the separation and concentration of aroma compounds due to their small molecular weight, high volatility, low concentration, and instability, as well as due to the impacts of the complex food matrix. Therefore, it is necessary to select an appropriate aroma extraction method with high extraction efficiency and minimal matrix interference that avoids the destruction of aroma compounds or the generation of new aroma compounds. With the increase in awareness of environmental protection, the development of green and sustainable extraction technologies has become an important trend in the field of food aroma analysis. Solvent-free extraction methods, due to their environmental friendliness, operational simplicity, and other advantages, have received increasing attention. For example, solid-phase microextraction (SPME) and stir-bar sorptive extraction (SBSE) can achieve efficient aroma extraction while reducing solvent usage, providing a more environmentally friendly option for food aroma analysis. Additionally, a stable, reliable, efficient, highly selective, and environmentally friendly extraction method should be used. The main two classes of aroma extraction methods (solvent-free and solvent extraction) are summarized in Figure 2.

#### 3.1.1. Solvent-Free Aroma Extraction Methods

Solvent-free aroma extraction methods include headspace (HS) extraction; the variances of adsorption extraction are also summarized. HS is the most popular sampling method with simple sample preparation for gas chromatography (GC) analysis. The principle of HS is that the coefficient between the released aroma and the food matrix in a closed system (a headspace extraction bottle with a 20 or 50 mL volume) maintains an appropriate temperature and waits for the volatile organic compounds to reach equilibrium between the sample matrix and the gaseous headspace substance [16]. There are two HS methods, static and dynamic HS, according to the sampling method used. For static HS, the equilibrium-released gas phase is directly injected into the GC injector using an automatic gas-tight syringe. The dynamic HS method, commonly called purge and trap (P&T), involves continuously passing nitrogen through the sample, enabling the aroma compounds passing through the carrier air to be absorbed by the sorbent trap [17]. Finally, the desorbed sample is injected into the GC injector by passing the heated carrier gas through the trap. Generally, the HS method is suitable for trapping compounds with low boiling points and high volatility. In addition, it is suitable for temperature-sensitive samples without complex pretreatment. HS is often used as a supplement to solvent extraction methods; for example, dimethyl sulfide is supplied as a highly volatile aroma-active compound in cold-pressed rapeseed oil using HS extraction for solvent-assisted flavor evaporation analysis (SAFE) [18,19]. HS is a simple, commonly used solvent-free method. It is easy to operate, non-destructive to samples, fast, eco-friendly, and can be automated. However, it has low sensitivity, and its extraction efficiency is affected by factors like the sample matrix, temperature, and equilibrium time. It is not efficient in extracting high-boiling-point or strongly adsorbed aroma compounds.

Solid-phase microextraction (SPME), the most widely used aroma method proposed by Belardi and Pawliszyn in 1989 [20], integrates sampling, concentration, and extraction into one step by extracting fibers. SPME has the advantages of simple, fully automated operation; being fast, efficient, sensitive, and solvent-free; and requiring only small sample amounts. Suitable fixed-phase materials are crucial for improving the adsorption capacity of analytes. Commonly used fixed-phase materials include polydimethylsiloxane (PDMS), divinylbenzene (DVB), carboxyalkene (CAR), carbon molecular sieves (Carboxen^®^), polyethylene glycol (PEG), and polyacrylate (PA) or their combinations, forming double-layer and three-layer fibers with better adsorption ability, resulting in a very wide range of adsorption polarities [21]. During the actual experimental process, a composite of polar and non-polar materials with a 30–100 μm polymer stationary phase was combined for the stationary phase to separate and enrich mixtures of various high-polarity compounds such as PDMS/DVB, CAR/PDMS, PEG/DVB, and DVB/CAR/PDMS [22]. To expand the application of SPME, the immersive approaches of direct-immersion solid-phase microextraction (DI-SPME) [23] and hollow-fiber membrane-protected solid-phase microextraction (HFM-SPME), which mainly focus on the detection of non-volatile compounds such as pesticides, polycyclic aromatic hydrocarbons, herbicide, and bisphenols, have been developed [24,25,26]. However, the drawbacks of SPME analysis include its low accuracy of aroma quantification, weak repeatability, and relatively few commercially available loaded polymer varieties. Thus, thin-film solid-phase microextraction (TF-SPME; 450 μm, PDMS, PDMS/DVB, and PDMS/HLB) [27] and SPME arrow (100~250 μm, PDMS, DVB/PDMS, and DVB/Carbon WR/PDMS) [24] were proposed to enable direct contact with the liquid sample, increase the contact area, and achieve better trapping of the high boiling compounds. This is achieved by attaching a thin-film-like extraction layer to a carbon mesh layer, which can be used for headspace or immersion extraction. TF-SPME has a larger extraction phase volume due to this thin-film geometry, resulting in higher extraction efficiency and enhanced sensitivity of the extraction process (even for non-volatile compounds) compared to the traditional SPME technique [28]. TF-SPME provides more effective agitation and increased extraction recovery with a shortened analysis time and has the advantage of high-throughput sample preparation when combined with an automatic 96-blade system. SPME arrow demonstrates better mechanical robustness and physical durability and offers a pre-drilled GC inlet septa, which mitigates issues pertaining to damage to the core during extraction and desorption, extending its service life. Overall, TF-SPME and SPME arrows have better adsorption capacity and efficiency and high sensitivity, but they are subject to challenges in terms of matching CTC equipment and injection port updates [29]. SPME is an important solvent-free technique. It does not require solvents, is easy to use, and affords quick extraction speed; in addition, its fibers are reusable for different samples. However, the fiber coating ages over time, its analyte adsorption capacity is limited, and matrix effects and contact time affect extraction efficiency.

A mono trap (DCC18, DSC18) has a porous monolithic structure consisting of pure silica and/or silica with activated carbon or graphite to provide a large surface area, which is chemically modified with octadecyl silane [30]. This enables it to effectively trap and adsorb volatile, semi-volatile, polar (neutral or acidic), and non-polar aroma compounds from liquid and gaseous matrices. There are disk and column types of mono trap (DCC18 and DSC18), which require specific desorption devices to release the absorbed aroma compounds and then inject them into the GC injection port. The use of a mono trap resulted in higher extraction concentrations of alcohols, aldehydes, acids, ketones, pyrazines, phenols, etc., from soy sauce than those obtained via the SPME method [31]. Additionally, dichloromethane and ethyl acetate can also be used as elution solvents to isolate aroma compounds from adsorbent materials without using any dedicated hardware [32]. The mono trap method is excellent for extracting trace aroma compounds, with high extraction efficiency, good selectivity, and stability, but it is costly and has specific regeneration complex and equipment requirements, which restrict its adoption.

Stir-bar sorptive extraction (SBSE), an aroma extraction technique based on the equilibrium distribution of target compounds between the aqueous solution (sample) and the stirring rod coating (extraction stage), was first proposed by Baltussen in 1999 [33]. The stirring rod was approximately 10–40 mm long and coated with a special material (a quantity 20–250 times higher than that used in SPME) to accelerate the distribution of the analytes between the substrate and the stationary phase coated on the stirring rod through stirring [34]. Currently, only three types of SBSE coatings, all commercialized by Gerstel, are available on the market, namely polydimethylsiloxane (PDMS), polyethylene glycol (PEG), and polyacrylate (PA) [35]. The stirring rod is placed into dedicated hardware to release the absorbed aroma compounds using thermal desorption after the removal of the residual solution on the surface of the stirring rod. SBSE can effectively extract volatile and semi-volatile compounds from aqueous samples, enabling sensitive determination. Furthermore, for complex food matrices, the absorbed SBSE can be eluted using low quantities of organic solvents. Overall, SBSE has advantages in aroma extraction involving relatively simple matrices such as water, distilled spirits, fruit juices, etc. [36,37]; however, it has certain limitations when used for viscous and complex matrix samples. SBSE has a large extraction capacity, a high enrichment factor, and a simple extraction process. However, the extraction time is long, the stir bar can be damaged by solid or high-viscosity samples, and the desorption process is complex.

Thermal desorption (TD), which is well-suited to the analysis of samples collected through diffusive sampling, uses a fixed adsorbent to capture gas molecules released from air, solid, or liquid samples in the adsorbent (the adsorption efficiency at 20 °C is 1000 L/g) [38]. Then, the gas-phase compounds overcome the adsorption force and escape from the solid surface (the adsorption efficiency at 250 °C is 10 mL/g) due to the increase in temperature, exhibiting good adsorption and desorption effects. The thermal desorption device can achieve a low temperature of −60~−100 °C through electronic refrigeration and with a fast flash evaporation heating rate. Moreover, automatic synchronous switching can be achieved through the electronic control of multiple valves to ensure that all samples enter the GC system without diffusion. Currently, porous polymers (Tenax^®^ TA, Markes, London, UK), graphitized carbon black (Carbopack™, Supelco, Bellefonte, PA, USA), carbon molecular sieves (Carboxen^®^, Sigma-Aldrich, Milwaukee, WI, USA), and inorganic adsorbents (silica gel, molecular sieves, and glass wool) are the four main sorbents used in TD [39,40]. Moreover, TD sampling is flexible and can be mobile according to the different application scenarios. It can simultaneously achieve the enrichment of in situ aromatic substances, such as the off-flavor samples from latrines [41], the aroma released during cooking stir-fried shredded potatoes [42], and the aroma released from the mouth or retronasal cavity [43]. An efficient solvent-free method, TD is highly efficient and good for high-sensitivity analysis, especially when combined with gas chromatography. However, it requires specialized equipment. High-temperature desorption may degrade some aroma compounds, and it is sensitive to sample preparation and desorption conditions.

#### 3.1.2. Solvent Aroma Extraction Methods

Solvent extraction is divided into three classes according to the extraction characteristics and solvent extraction (LLE, LSE, and ASE), distillation (SDE, VDE, SAFE), and solvent elution (SPE) methods. Solvent extraction, including liquid–liquid/solid extraction, was first proposed by Bucholz in the early 19th century. Based on the principle of similarity compatibility, the target substance was extracted by utilizing the solubility difference in the compound between different solvents, which can be understood as the aroma compounds transferring from one phase (liquid or solid) to another (liquid) phase [44]. It has been widely used in aroma analysis due to its many advantages, such as a wide range of compounds for selection, simple operation, and the wide variety of available solvent choices [45]. Currently, dichloromethane, diethyl ether, pentane, hexane, methanol, acetonitrile, acetone, and chloroform are the extraction solvents most often used in isolating aromas from meat, wine, Baijiu, Huangjiu, drink, fruit, milk, seafood, etc. Solvent extraction can simultaneously extract volatile and semi-volatile compounds. Furthermore, to expand the range of extraction of aroma compounds, mixtures of solvents with different polarities, such as pentane/dichloromethane (2:1), diethyl ether/dichloromethane (1:1), and hexane/methanol (1:1), have been used [46]. However, in recent years, due to the high volume of solvents used and carcinogenic risk, researchers have been investigating miniaturized extraction techniques to reduce solvent usage without compromising extraction efficiency and sensitivity, for example, liquid–liquid microextraction (LLME) and single-drop microextraction (SDME), involving the distribution of analytes between a small amount of water-immiscible solvent and an aqueous phase containing the target analyte [47]. Additionally, several different SDMEs have been derived, like dispersive liquid–liquid microextraction (DLLME), solidified floating organic drop DLLME, vortex-assisted DLLME, and air-assisted DLLME [48]. These multiple solvent extraction methods have been widely applied in the aroma extraction of alcoholic beverages, fruits, meat products, etc.

Accelerated solvent extraction (ASE) is an automated and rapid method for extracting aroma compounds from solid or semi-solid food using organic solvents under high-pressure and high-temperature conditions [49]. This method is mainly derived from classic isolation techniques, such as Soxhlet extraction, which are tedious and time-consuming. The solvent (dichloromethane, methyl tert-butyl ether, diethyl ether, petrol puriss, acetone, etc.), pressure (500~3000 psi), temperature (50~200 °C), and extraction time (5–15 min) are the main parameters of ASE [50]. A high temperature can weaken the interaction forces generated by van der Waals forces, target molecules, hydrogen bonds, and dipole attraction at the active sites of the sample matrix. The solubility of liquids far exceeds that of gases, thus increasing the extraction pressure and the boiling point of the solvent. The advantages of ASE are the low volume of organic solvent required, rapidity, minimal matrix influence, a high recovery rate, and good reproducibility. Its disadvantage is that the pressure of organic solvents is relatively high, and this creates high safety requirements for equipment to prevent disasters such as fire and explosion [49]. Due to ASE’s powerful extraction capability, its extraction parameters should be optimized to avoid the effects of resin and pigments. Moreover, high-temperature extraction can easily lead to the degradation of thermosensitive compounds. Thus, to overcome these drawbacks, the ASE high-vacuum instrument (ASE-HV) was developed to extract aroma compounds at a low temperature [51].

Simultaneous distillation extraction (SDE) is an extraction technique first proposed by Nickerson and Likens in 1964 [52]. It is based on the boiling and thorough and simultaneous mixing of the aqueous and solvent phases, resulting in these phases refluxing in the condenser, combining distillation and extraction into one step. This technique has usually been considered superior to classical distillation or solvent extraction. SDE can trap a large number of high-boiling compounds in high concentrations with low extraction solvent volumes (50 mL), but it is not very efficient in trapping low-boiling compounds with higher volatility [53]. The advantage of SDE is the high extraction recovery rate for chemical components with medium-to-high boiling points. In addition, SDE is suitable for aroma extraction from soup or cooking dishes. For example, smoke-cured bacon was extracted using SDE for 4 h in our previous work [54], which obtained a reconstitute sample with high-similarity aroma profiles. For essential oil extraction, the SDE method proves to be a suitable option for obtaining extracts free from cuticular waxes or chlorophylls [55]. However, due to its long extraction time at high temperatures, the retention efficiency of thermosensitive and highly volatile compounds was relatively low, and some thermal processing by-products were also derived. Therefore, an instrument for simultaneous vacuum distillation extraction (VDE) was proposed to overcome these disadvantages [56]. VDE performed at lower pressures is suitable for the separation and extraction of liquid mixtures with thermosensitive and high-boiling point aroma compounds [57]. VDE can be performed at low temperatures, thus avoiding the decomposition of the product during the extraction process. Moreover, the vacuum environment creates an ideal dynamic process for promoting the dissolution and diffusion of effective components.

Solid-phase extraction (SPE), which was first introduced in the mid-1980s [58], is based on the interaction between the adsorption of an analyte on a particular material and elution by solvents of different polarities. This process can efficiently enrich target compounds and remove interfering factors like fats, proteins, pigments, macromolecular substances, and alkane compounds, improving the accuracy and sensitivity of analysis. SPE has the advantages of simple operation, fast speed, and the capacity to remove interference from large-molecular proteins and fats. However, SPE exhibits limitations in the isolation of fatty acid ester aroma compounds. Short-chain fatty acid esters exhibit significant polarity variations, while long-chain fatty acid esters are weakly polar or non-polar. Thus, the extraction efficiency of these fatty acid ester compounds is significantly influenced by the adsorbent’s polarity. Polar adsorbents (e.g., C18 silica gel) demonstrate poor retention capacity for non-polar fatty acid esters, leading to their potential loss during the elution process. For instance, in lipid-based food analysis, using polar adsorbents may reduce the recovery rate of non-polar fatty acid esters by over 50%, compromising the accuracy of aroma profiling. Therefore, it is necessary to optimize adsorbent selection according to the polarity of target fatty acid esters and consider mixed adsorbents or adjusted elution conditions when necessary.

SPE matrices are mainly divided into three categories according to the absorption filler materials: silica gel matrices (a chemical synthesis reaction between chlorine or alkoxy groups and silica gel forms a bonded silica gel with different functional groups, such as C18 (octadecyl), C8 (octyl), etc.), inorganic matrices (inorganic oxides, such as active silica, alumina, Florisil, etc., and graphitized carbon inorganic substances), and organic polymers (most are styrene divinylbenzene copolymers (PS-DVBs), polymethyl methacrylate (PA), styrene divinylbenzene vinyl ethylbenzene copolymers (PS-DVB-EVBs), and styrene divinylbenzene vinyl pyrrolidone (PS-DVB-NVP), among others). The silica gel and inorganic matrices are the most popular types of matrices in aroma extraction. During the application, the reverse-phase, positive-phase, and ion-exchange types of SPE are divided according to the sorbents utilized [59]. Reversed-phase SPE predominantly employs hydrophobic materials such as C18 or C8 bonded silica for extracting non-polar to moderately polar compounds, while normal-phase SPE utilizes polar adsorbents like unmodified silica gels [60]. However, positive-phase SPE is exactly the opposite of normal-phase SPE; during positive-phase SPE, the liquid sample is passed through a column or disc containing a stationary phase. Fixed phases have specific chemical or physical properties, and analytes are retained in the stationary phase due to adsorption, distribution, or other forces. Subsequently, the analyte is eluted from the stationary phase using an appropriate elution solvent, achieving aroma separation from the sample matrix. Overall, SPE uses low quantities of solvents, which can significantly remove impurities and enrich the aroma compounds. SPE has been used to effectively identify key aroma compounds like 4,5-dimethyl-3-hydroxy-2,5-dihydrofuran-2-one and 2-methyl-3-furanthiol from complex matrices [61,62].

Solvent-assisted flavor evaporation (SAFE) is a vacuum distillation technique developed by Engel in 1999 for the direct separation of aroma compounds from the solvent extracts and aqueous foods under high-vacuum conditions (1 × 10^−3^~1 × 10^−4^ Pa) [63]. SAFE can effectively isolate the volatile and semi-volatile compounds from non-volatile matrices, reducing the loss of thermosensitive volatile components during the extraction process, which preserves the original flavor of the extracted material. Model solution exaction, a common way to investigate the extraction method performance, has confirmed that SAFE can achieve high yields of aroma compounds from fatty matrices (50% fat). Moreover, the aroma compounds of Chinese butter hotpot seasoning were also isolated using SAFE, proving that it was suitable for foods with fatty matrices [64]. Currently, SAFE is an important part of the sensomic approach. During the SAFE process, the solvent flow needs to be adequately controlled to form droplets to ensure the complete vaporization of the solvent or aqueous solution. Furthermore, the temperature (40~60 °C) of the manual valve section could be easily reduced due to the heat absorption during gasification, especially for samples with a high-fat content, which are prone to condensation and blockage. Moreover, the manual valve of the SAFE equipment leads to suboptimal yields and the risk of contamination of volatile isolates with non-volatile compounds. Therefore, Schlumpberger [65] invented an automated SAFE (aSAFE) device to address the following issues: the complex extraction process, the time-consuming addition of liquid nitrogen, and the adequate heating of the manual valve section. The aSAFE provides substantial advantages in terms of the yield of volatile compounds and the risk of transfer of non-volatile compounds into volatile isolates and also reduces the labor required.

### 3.2. Qualitative Analysis of Aroma Compounds

#### 3.2.1. Chromatographic Separation Technology

**Figure 3:**
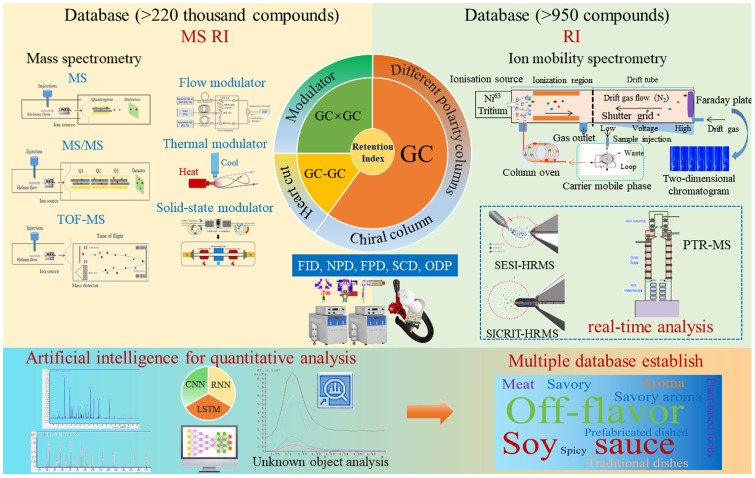
Methods of aroma qualitative analysis.

After the extraction and enrichment of aroma compounds from foods, further qualitative and quantitative analyses are needed to determine the main aroma compounds and to identify the key odorants. Traditional aroma analysis relied on GC-FID and GC-MS. Recent innovations (post-2010) include GC-IMS for rapid screening and GC×GC-TOF-MS for resolving complex matrices [66] (Figure 3). Additionally, there are other aroma analysis methods, such as electronic nose, nuclear magnetic resonance, and infrared spectroscopy. These methods exhibit broad-spectrum responsiveness to diverse aroma compounds, with their discrimination mechanisms primarily rooted in the molecular architecture and functional group characteristics of compounds. They are primarily employed for the following purposes: (1) to differentiate between samples, (2) to facilitate rapid cluster analysis, and (3) to establish quantitative frameworks for freshness assessment, process monitoring, high-throughput quality evaluation, etc. Therefore, these methods are not discussed in this review.

GC, invented by Martin and James in 1952, has become the most important and widely used technique for aroma analysis [67]. Helium or hydrogen serves as the mobile phase in GC, enabling the efficient separation of complex aroma mixtures based on their volatility and polarity. When an analyte is injected into the chromatographic column, the distribution coefficients between the gas phase (aroma compounds) and the stationary phase are different due to the combined action of three interaction forces (dispersion force, dipole force, and hydrogen-bonding force) on each component in different polar chromatographic columns [68]. The non-polar column separation is based on the boiling point of aroma compounds, while the polar column separation is based on their boiling point and polarity. GC is the most fundamental technique for aroma analysis (Figure 2). Different polarity columns (the separation of aroma compounds based on their boiling point, polarity, structure, etc.) and chiral columns (the separation of optical isomers) were developed to separate the aroma compounds from aroma extracts. However, multidimensional gas chromatography (MDGC), which has excellent separation efficiency, was developed for complex volatile and semi-volatile samples based on two chromatography columns with different polarities to overcome the co-elution effect [69,70]. GC is a commonly used separation technique in food aroma analysis as it exhibits high efficiency and sensitivity in the separation of volatile and semi-volatile aroma compounds. Samples are vaporized and then carried by the carrier gas through the chromatographic column. However, with the increasing requirements for food aroma analysis, the limitations of traditional GC technology with respect to co-elution with complex samples have been gradually revealed. To better separate complex volatile and semi-volatile samples, multidimensional chromatography techniques have emerged. Multidimensional chromatography techniques, such as GC-GC (two-dimensional GC, where two chromatographic columns are connected in series to achieve more efficient separation through different separation mechanisms) and GC×GC (comprehensive two-dimensional GC, which has a higher resolution and peak capacity and can more precisely separate complex mixtures), play an increasingly important role in food aroma analysis. They can further separate complex aroma mixtures, providing a significantly higher separation ability.

At present, heart-cut (Dean’s witch system, GC-GC) and comprehensive two-dimensional gas chromatography (GC×GC) are two representative two-dimensional gas chromatography techniques that play an important role in food aroma analysis [66]. GC-GC is mainly focused on the known compounds to determine the cutting time (the time point at which a part of the fractions is separated by the first-dimensional chromatographic column and then is transferred to the second-dimensional chromatographic column for further separation), and only a part of the unknown co-elution analytes is sent to the second chromatographic column for separation using the modulator. The second-dimensional (2D) chromatographic column has similar dimensions (length, inner diameter, peak capacity) to the first-dimensional (1D) chromatographic column, but the stationary phase (or comparison) is different. Heart-cut GC-GC avoids cryogenic modulation, simplifying instrumentation but limiting throughput compared to GC×GC [71]. However, the GC×GC is a continuous heart-cut system for all fractions in the first-dimensional chromatographic column, which is conducted using the modulator and is typically performed within a time window smaller than the one-dimensional peak width [72]. The second chromatographic column is very short and has a limited peak capacity, as the second separation needs to be completed within the time range of the transfer window (modulation time). The modulator is the key link between the 1D and 2D columns to achieve multidimensional chromatographic separation. There are two main types of modulators: the flow modulator (which blows the sample onto the 2D column using a high-flow carrier gas and is based on the complex gas circuit) and the thermal modulator (which uses a refrigerant cold spray and has solid-state control). The thermal modulator is the most popular due to its high efficiency in trapping and concentrating aroma compounds by employing oscillating temperature gradients, and its release process transfers the sample components to the head of the 2D column as a narrow pulse [71]. The solid-state thermal modulator utilizes semiconductor refrigeration and direct heating to achieve rapid modulation, thus completely eliminating the use of liquid nitrogen and other refrigerants as in traditional GC×GC and significantly reducing the difficulty and operating cost [73]. Regardless of whether the traditional GC or multidimensional chromatography (GC-GC or GC×GC) separation is used, calculating the retention index of compounds based on the reference of n-alkanes is still an important qualitative method. Due to the significantly high separation capacity of GC×GC, specialized data software like Canvas (2.6 version, J&X technologies) and chromaTOF® (4.40 version, LECO Instruments) were developed, which can resolve more than 100,000 peaks at once.

The combination of GC with the mass spectrum and olfactory system (GC-MS/O) remains the most widely used classical technology for the identification of key aroma compounds in foods. By applying this method, more than 226 key aroma compounds have been identified from 270 different foods [2,74]. In recent years, due to it is powerful separation ability, GC×GC has been frequently applied for decoding the aroma composition of different foods, such as ham, tea, *Zanthoxylum bungeanum*, grilled lamb shashliks, etc. [75,76,77]. More than 200 volatile compounds and over 100 aroma-active compounds have been identified using the GC×GC technique.

Liquid chromatography (LC) is also an important separation technique in aroma analysis. It separates substances based on the difference in their distribution coefficients between the stationary phase and the mobile phase. LC is suitable for separating polar and less volatile aroma compounds. Its advantages include high separation efficiency and a wide variety of stationary and mobile phase options. However, it has some limitations, which include its relatively long analysis time and less effective analysis of highly volatile compounds compared to GC. In summary, chromatographic separation technology is essential for food aroma analysis. To better understand food aromas, we need to continue developing chromatographic techniques and column materials. Researchers should also combine different chromatographic methods to improve the accuracy and comprehensiveness of aroma analysis, which will help us understand the relationship between aroma compounds and food quality.

#### 3.2.2. Instrumental Detector for Aroma Identification

Mass spectrometry is by far the most important analytical technique in the field of aroma identification based on MS databases. After gasification and chromatographic column separation, the neutral aroma molecules are ionized by an ion source and then detected by the mass spectrometer. Electron ionization (EI) is the most common ion source. In the process of electron ionization, electrons generated by a filament accelerate at a speed of 70 electron volts (eV), knocking out the electrons from the aroma molecules and producing molecular ions (free radical cations). This high-energy ionization produces unstable molecular ions, and the excess energy is lost through fragments [78]. The chemical bonds break, leading to the loss of free radical cations or neutral molecules, resulting in rearrangements. Finally, ions of different masses are produced. However, the number of ions depends on the structure, filament acceleration, functional group, EI voltage, etc. The advantages of EI include its high ionization efficiency and sensitivity, which can provide rich structural information and are therefore regarded as the fingerprint of aroma compounds. Due to the widespread application and good reproducibility of EI, almost all mass spectra of standard compounds have been obtained via EI. However, the technique also has its limitations in detecting thermally unstable and difficult-to-volatilize compounds because these compounds may decompose or change under EI conditions, thereby affecting the accuracy of the analysis results. To solve this problem, a chemical ionization (CI) source was developed by conducting chemical reactions at the ion source with reaction gases such as methane, isobutane, and ammonia to produce molecular ions [79,80]. However, positive chemical ionization (PCI) generating protonated molecular ion peaks or complex ions to confirm the molecular weight and negative chemical ionization (NCI) sources with high sensitivity and specificity were divided, respectively. CI is the result of one or several competing chemical reactions. In the process of CI, multiple different types of chemical reactions between the reaction gas and the sample molecules occur simultaneously, and these reactions compete with each other. Therefore, the sensitivity of CI sources largely depends on the experimental conditions. In addition to electron energy and electron current, the type, purity, and pressure of the reaction gas and the temperature of the ion source affect the sensitivity of the CI mode.

A quadrupole is the most common type of mass analyzer. It is a scanning instrument that changes the voltage to allow only ions with a specific *m*/*z* to pass through the quadrupole in a stable trajectory to reach the ion detector. The quadrupole instrument has two different working modes: SCAN (which obtains all ions within a certain quality range and is suitable for the identification of unknown substances, method development, and qualitative and quantitative analyses of high-concentration analytes) and SIM (which only collects selected ions representing the target compound and is suitable for trace analysis due to its higher sensitivity). After being separated by the mass analyzer based on the *m*/*z*, the ions reach the ion detector, where the signal is amplified by an electron multiplier (used for most low-resolution mass spectrometers (LRMSs)) or a multi-channel plate (used for most HRMS instruments). Finally, the signal is recorded by the acquisition software (MassHunter Qualitative DA Software 12.0) on the computer to plot the chromatograms and mass spectra for each data point. In summary, single quadrupole mass spectrometry (GC–MS) has a strong quantitative ability, simple structure, and low maintenance cost. Triple quadrupole mass spectrometry (GC-MS/MS) retains the quantitative ability of GC-MS while achieving enhanced qualitative ability, resulting in higher maintenance costs. GC–MS/MS demonstrates not only high-sensitivity and enhanced quantitative and qualitative abilities but can also use the multiple reaction monitoring (MRM) mode to monitor specific precursor–product ion pairs, effectively eliminating matrix interference and improving the detection accuracy of trace aroma compounds in complex samples [81]. GC-TOF-MS (gas chromatography–time-of-flight mass spectrometry) combines high speed and resolution, making it ideal for untargeted profiling [72]. GC–TOF–MS is the fastest mass spectrometry technique with a high resolution, which helps distinguish between qualitative and *m*/*z* approximate ions [82]. GC–TOF–MS features high resolution and fast scanning, enabling the acquisition of a large amount of mass spectrometry information in a single analysis, which is conducive to the comprehensive qualitative analysis of complex aroma components. However, it has a relatively high instrument cost and requires the operators to have relatively high technical skills. GC-QTrap incorporates MRM (multiple reaction monitoring), SRM (single reaction monitoring), neutral loss, and multi-level cascade, making it very suitable for the structural analysis of unknown compounds, but its resolution is lower than that of GC–TOF–MS [83].

Gas chromatography–ion mobility spectrometry (GC–IMS), which provides the strong two-dimensional separation capability of GC and high-resolution IMS, has been popular for food aroma analysis and aroma perception during food oral processing [84]. IMS can detect trace amounts of aroma compounds at the ppb level and exhibits a fast response and high sensitivity at normal pressure. It separates ions based on their mobility in an electric field in the nitrogen carrier gas (Figure 2) [85]. Injection occurs through a six-way valve system, which can trap 200 μL of a headspace gas sample injected into the GC. The GC section can then separate the aroma compounds with a high flow rate from 1~200 mL/min within 30~80 °C. The ionization source (^3^H, 300 MBq) and drift tube with a high drift voltage (6.5 keV) comprise a core part of the IMS system. After the gas phase is transferred to the ionization region by a carrier gas, the neutral aroma molecules are subsequently ionized by the ion source to form various fragment ions with different drift times in the separation region. These fragment ions travel under the influence of an electric field and have different mobilities, resulting in different drift times, providing information about the type and concentration of the analytes. Commercial GC-IMS systems are widely adopted for rapid aroma profiling in food quality control. FlavourSpec^®^ (G.A.S., Dortmund, Germany) has conducted extensive research on authenticity identification, quality identification, and fingerprint molecular analysis based on aroma analysis [86,87]. The aroma compounds detected by GC-IMS can be identified based on the retention indexes calculated according to the laboratory analysis view (VOCal, 0.4.03 version) software by comparing them with C3–C9 n-ketone reference mixtures. Moreover, identification via IMS is based on a database of standard compounds (>950 compounds).

GC-MS/O (gas chromatography–mass spectrometry/olfactometry) integrates chemical and sensory analyses, enabling simultaneous compound identification and odor description [88]. The integration of gas chromatography, mass spectrometry, and human olfactory perception (GC-MS/O) into the instrument originated from two technological revolutions, namely the gas chromatograph olfactory (GC-O) and mass spectrum [89] technologies. The greatest challenge to this technique is that the mass spectrometer works under vacuum conditions, and the olfactometric detector works under atmospheric pressure conditions. Therefore, a restrictor was developed to increase the pressure drop between the interface and the flow splitter; in addition, careful selection of the flow rates of the carrier and auxiliary gases can overcome the pressure difference [88]. During GC-MS/O analysis, aroma-active compounds can be screened simultaneously and identified, along with their odorant description. GC×GC–MS/O was developed to obtain more aroma-active compounds and decode the unknown compounds (which are detected by GC-O but could not be identified by MS) [73]. Subsequently, it was applied to characterize the key aroma compounds in soy sauce, beef flavoring, breast milk, etc. [89,90]. Currently, the OSME (odor-specific magnitude estimation), CHARM (combined hedonics of aromatic response measurement), and AEDA (aroma extraction dilution analysis) are the most popular methods of identifying and selecting aroma-active compounds from food samples. OSME can obtain the RI, aroma quality, and perception intensity of aroma-active compounds to determine aroma-active compounds and their significance [91]. AEDA [92] and CHARM [93] are both based on measuring an aroma threshold determined through a series of dilutions. Aside from traditional means of obtaining aroma-active compounds, the taste perception-enhancing odorants can be screened via the gas chromatography/olfactometry-associated taste (GC/O–AT) method [94]. By applying this method, several saltiness-enhancing odorants were identified in soy sauce, ham, etc. [95,96].

Although detection instruments can separate and identify aroma compounds, the concentration of individual compounds cannot directly reflect their contribution to the overall aroma profile. Therefore, the odor activity value (OAV, the ratio of concentration to the threshold value) has become a key indicator for evaluating and screening key aroma compounds [97]. Generally, the compounds with OAV ≥ 1 are considered potential aroma contributors. For example, 3-octanone (OAV = 1657), 1-octen-3-ol (OAV = 4562), and octanal (OAV = 9324) were determined to be the potential key odorants contributing to the “mushroom aroma” of Flammulina velutipes via the application of the GC-MS/O and AEDA techniques combined with an OAV calculation [98]. Based on the OVA results, 2-methoxy-phenol, ethyl acetate, 3-methyl-1-butanol, 3-methyl-butanal, methional, dimethyl trisulfide, and dimethyl disulfide were identified as the potential key odorants in soy sauce [99]. The application of OAV not only helps identify characteristic aroma markers but also provides a theoretical basis for optimizing food processing.

Other detectors, including FID, NPD, FPD, and SCD, have been applied in aroma identification. FID has two electrodes, one of which is a nozzle for flame combustion, and the other is used to collect ions in the flame after applying a polarization voltage. When the components enter the flame, the current recorded by the collection electrode increases. The current is amplified to form a chromatogram. The principle of the NPD is that there is a rubidium bead heated by a high current above the nozzle of the detector, and the alkali metal salt (rubidium bead) escapes a small number of ions when heated [100]. A polarization voltage of −250 V is applied to the rubidium bead, forming a direct current electric field with the cylindrical collecting electrode. The small number of escaped ions move directionally under the action of a direct-current electric field, forming a small current that is collected by the collection electrode, which is called the base current. When organic compounds containing nitrogen or phosphorus flow out of the chromatographic column, thermal ionization reactions occur around the rubidium bead, greatly increasing the ionization degree of the alkali metal salt (rubidium bead). The generated ions move in a directional manner under the action of a direct-current electric field, forming a small current that is collected by the collection electrode. The signal is then amplified by a microcurrent amplifier and processed by an integrator to achieve qualitative and quantitative analyses.

Flame photometric detectors (FPDs) are mainly for the detection of phosphorus- or sulfur-containing aroma compounds. The core purpose of an FPD is to quantitatively analyze the characteristic spectra emitted by organic compounds containing phosphorus or sulfur during combustion in hydrogen-rich flames [101]. This analysis method is based on the proportional relationship between the content of the measured component and the intensity of the characteristic light. Through the combination of a filter and a photomultiplier tube, the optical signal is ultimately converted into an electrical signal, thereby achieving a quantitative analysis of the measured component. SCD is mainly used to detect sulfur-containing compounds when these compounds burn at high temperatures to form sulfur monoxide (SO), which then reacts with ozone (O_3_) to form excited-state sulfur dioxide (SO_2_) [102]. When the latter decays to its ground state, it emits a characteristic blue spectrum (wavelength 280–420 nm). After passing through a filter, this light wave is detected by a photomultiplier tube; thus, sulfur is detected.

Each detector for aroma identification offers unique capabilities; none can provide a complete solution on their own. The diversity of detector principles and levels of performance necessitates a strategic approach. By integrating various detectors based on the specific food aroma analysis requirements, such as the complexity of the sample and the target analytes, we can enhance the accuracy and reliability of our results. Additionally, advancements in detector technology should be complemented by parallel developments in sample preparation and data analysis techniques. This holistic approach will not only improve our understanding of food aroma but also drive innovation in the food industry, enabling the creation of products with enhanced sensory qualities. As the field continues to evolve, continuous research and development in instrumental detectors will remain crucial for unlocking new insights into the complex world of food aromas.

In addition to traditional offline detection methods, real-time aroma analysis technology has shown unique advantages in the online monitoring of food processing. Proton transfer reaction high-resolution mass spectrometry (PTR-HRMS) ionizes volatile organic compounds through a proton transfer reaction in combination with high-resolution mass spectrometry to achieve real-time monitoring [103]. The detection limit can reach the ppb level, making it suitable for the dynamic tracking of key aroma components such as ethanol and esters during fermentation or the oral processing of different foods [104,105]. Electrospray spray desorption ionization high-resolution mass spectrometry (SESI-HRMS) uses micro-droplets generated via electrospray to extract volatile matter on the surface of the sample and directly carries out mass spectrometry analysis [106]. Without complex pre-treatment, it enables the online monitoring of the aroma release law in baking, cooking, and other processes. Surface desorption atmospheric pressure chemical ionization high-resolution mass spectrometry (SICRIT-HRMS) uses atmospheric pressure chemical ionization technology to achieve the in situ real-time detection of volatile components on the surface of solid food. By applying SICRIT-HRMS in real-time monitoring analysis, thermal processing (pasteurization) was found to significantly reduce the content of tangeretin (a citrus flavonoid) and riboflavin (riboflavin), while high-pressure processing better preserved the volatile characteristics of fresh orange juice [107]. These technologies provide support for optimizing food processing parameters, predicting shelf life through real-time data feedback and significantly improving production efficiency and quality control accuracy.

#### 3.2.3. Artificial Intelligence for Qualitative Analysis

Due to the variety of powerful isolation methods and detectors, aroma analysis produces complex chromatograms, resulting in time-consuming data processing. Machine learning, an advanced data analysis technique, provides new possibilities for improving the efficiency and accuracy of chromatographic analysis by learning patterns and prediction patterns from large amounts of data [108]. The application of machine learning in chromatographic analysis is first reflected in data processing and preprocessing. The data generated via chromatography often contain issues such as noise, baseline drift, and overlapping peaks, which can affect the accuracy of the analysis results. Machine learning algorithms, particularly deep learning techniques, can be used to remove noise, correct baselines, and resolve overlapping peaks. For example, convolutional neural networks (CNNs) can be used to identify and separate various component peaks in chromatograms, improving the signal-to-noise ratio of the signal [109]. Sequence models, such as recurrent neural networks (RNN) [110] and long short-term memory networks (LSTM) [111], can handle the time series characteristics of chromatographic data and predict and compensate for dynamic changes in the chromatographic process. Graph Neural Network (GNN) is an effective deep learning model specifically designed to handle a graph data structure, with a learning node core and edge feature representations through message-passing mechanisms [112]. The core advantage of GNN in flavor classification lies in its efficient modeling of hierarchical relationships and global information through a graph structure, making it especially suitable for complex constraint problems in food ingredient analysis. It has been proven to predict the aroma characteristics of aroma molecules by encoding the graph structures. Additionally, food ingredients have complex hierarchical structures, and traditional methods struggle to handle such unstructured relationships [113]. The framework exhibits a robust cross-dataset generalization capability, enabling its adaptation to heterogeneous data formats through graph-structured transfer learning (e.g., subgraph matching techniques). By integrating cross-category transferability with multimodal learning compatibility, this approach demonstrates enhanced versatility and significantly enhances its practical utility in real-world scenarios. Based on these artificial intelligence processing techniques, many aroma characteristic databases could be established, including an off-flavor database or databases for specific dishes or plant-based food aromas (Figure 2), enabling the quality control and improvement of distinctive food aromas.

Aroma detectors are fundamental in food aroma analysis. Although current detectors have their own merits, the complexity of food aroma composition calls for further development. More advanced detection principles and integrating improved detector miniaturization and portability should be the focus. Better data interpretation methods also need to be developed in parallel with detector advancements. By doing so, we can not only achieve more accurate and efficient aroma identification but also better meet the diverse needs of the food industry, such as rapid on-site detection and real-time quality control of food products. This will further promote the development of the food aroma analysis field and enhance our understanding of the complex relationship between food aroma and food quality.

Through the above-mentioned qualitative analysis methods, we can preliminarily determine the types of aroma compounds in food. Qualitative analysis is a crucial foundation for food aroma analysis. It helps us identify the aroma components present in food, paving a path for future research. However, merely knowing the types of aroma compounds is insufficient. To gain a deeper understanding of the composition and content of food aromas, quantitative analysis is essential. Quantitative analysis can accurately measure the concentration of various aroma compounds, which is of great significance for evaluating food quality and flavor characteristics and studying the interactions between aroma compounds. Next, several quantitative analysis methods commonly used for aroma compounds will be introduced in detail.

### 3.3. Quantification Analysis of Aroma Compounds

**Figure 4:**
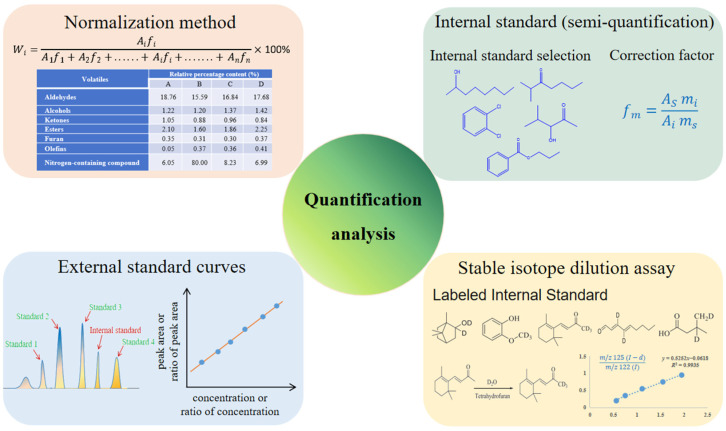
Aroma quantification analysis methods.

The goal of quantification analysis is to accurately detect the concentration of various aroma compounds in food samples, requiring an accurate determination of the peak area or peak height based on different detectors such as MS, FID, and SCD. At the same time, the most suitable quantitative method should be selected according to various factors, such as laboratory conditions, the sample amount and properties, and the aroma extraction method used. In addition, the selection of internal standards has a significant impact on quantitative results due to differences in the response signals of different types of compounds. The four main quantitative methods applied to aroma compounds are normalization, the internal standard method, the standard curve method, and the stable isotope dilution analysis method (Figure 4).

#### 3.3.1. Normalization Method

The normalization stage of quantitative analysis is performed by calculating the sum of the peak areas (except for the solvent peak) of all aroma compounds as 100% to determine the relative percentage of the content of each compound. This method is suitable for compounds that can completely flow out of the chromatographic column and trigger reaction signals in the detector, thereby forming identifiable peak samples in the chromatogram. Using this method, the dominant aroma compounds and their relative contents can be determined quickly, as can their quantitative relationship (ratio) among different samples [114]. To overcome variance in the signal responses, correction factors for the aroma compounds are used in normalization quantification. The advantage of this method is that it is simple, quick, and unaffected by the injection volume/amount. However, it is rarely used now because the specific concentration of each aroma compound cannot be accurately measured to control the food aroma quality.

#### 3.3.2. Internal Standard Method

The internal standard method is an indirect way to determine the relative concentration of the aroma compounds in food by adding internal standard compounds. The content of the target analyte is calculated based on the ratios of the peak area and concentration of the target compound to the internal standard; correction factors for the target compound should be considered to correct the signal response errors of different aroma compounds. The internal standard method can be used not only for relative quantification but also for absolute quantification when the accurate concentration of the internal standard and relevant correction factors are known [115]. Selecting suitable internal standard compounds is key to accurately calculating the relative content of analytes. First, the internal standard should not naturally exist in the food samples, and it should be possible to separate it from the aroma compounds in the sample without co-elution effects. Secondly, the internal standards should have similar physical and chemical properties (molecular weight, boiling point, and structure) to the analyte and must be able to completely dissolve in the extract solution or release completely during extraction; for example, a solid sample extracted via SPME/HS. Traditionally, a single internal standard was selected, such as 2-methyl-3-heptanone, 1,2-dichlorobenzene, 2-octanol, 4-hydroxy-4-methyl-2-pentanone, propyl benzoate, etc. [116,117,118]. However, multiple internal standard compounds have been used to handle different types of aroma compounds present and improve the accuracy of quantification results [62]. The internal standard method is commonly used when the content of sample components is too high or when it is difficult for all components in the mixture to peak. The advantage of the internal standard method is that external conditions have a relatively small effect on the quantitative results, meaning that the analysis accuracy is high. Due to differences in chromatographic conditions and injection volumes, errors caused by internal standard methods are smaller than those caused by external standard methods.

#### 3.3.3. External Standard and Internal Standard Curve Methods

The external standard method is also known as the standard curve method or direct comparison method. By plotting a standard curve, the concentration of the target aroma compound can be accurately determined. This is performed via serial dilution of a solution containing a mixture of standard aroma compounds; GC-MS is used to analyze the sample [119]. However, the aroma mixture’s standard solution should be divided into several groups according to their retention time and relative concentration to ensure the interference of compounds with similar peak times and significant peak area variances. The advantages of this method include its ease of use, suitability for analyzing a large number of samples, and ability to directly reflect the relationship between the concentration of the target compound and the response value. Its disadvantages include the strict requirements for experimental conditions. The processes of treating samples and standard solutions need to be highly consistent; otherwise, errors are likely to occur. The matrix effect may also affect the accuracy of the results. There are three steps to obtaining the standard curves: (1) first, the preparation of the standard aroma solution mixture sample at different concentrations (over five gradients) in two- or three-fold dilution; (2) the injection of equal volumes under the same conditions as the tested samples and the measurement of the peak height/peak area of each aroma compound; and (3) plotting the standard curves using the peak height/area and actual concentration to obtain the standard curve of each aroma compound. The concentration of the analytes can be calculated based on these curves. However, the internal standard compounds were added at the same volume and concentration as the standard solution during aroma extraction to overcome systematic errors or differences between samples and standards during the extraction process; this is the so-called internal standard curve method [120]. Each standard curve was carried out by plotting the response area ratio of the standard compounds to different internal standards against their concentration ratio. The selective ion monitoring (SIM) model of mass spectrometry was always used for the accurate quantification of aroma compounds.

#### 3.3.4. Stable Isotope Dilution Analysis (SIDA)

Stable isotope dilution analysis (SIDA) involves the use of a stable isotope-labeled compound with known mass and abundance as a diluent to the sample. It allows for the robust quantitative analysis of key aroma compounds by determining changes in position and abundance before and after the stable isotope reaction [121,122]. Stable isotope (^13^C and ^2^H)-labeled chemical compounds are molecules with almost identical structures, chemical properties, and chromatographic and mass spectrometry behaviors to the analyte. The molecular structure of this compound is very similar to the analyte, which can effectively eliminate ionization changes and matrix effects, thereby improving the accuracy and reliability of the analysis. During quantification, the calibration factors (R) of each aroma compound should be determined in mixtures of equal amounts of unlabeled odorants and corresponding labeled standards with ratios of 3:1 to 1:3 (by weight) via mass chromatography. For example, the known amounts of labeled (*m/z* 139) and unlabeled linalool (*m/z* 137) in different mass ratios (1:3 to 3:1) of the MS response area value were plotted to obtain the slope representing an R-value of 0.86 [9,121]. However, there are several challenges associated with the SIDA method: (1) its high cost, (2) its instability and difficulty with preservation, and (3) that high-resolution GC-MS is needed for quantification. Due to these challenges, most laboratories use the standard curve method for quantification.

## 4. Flavoromics

Flavoromics, an emerging interdisciplinary field at the intersection of food science and analytical chemistry, has revolutionized our understanding of food flavors by integrating advanced aroma analysis techniques with comprehensive, data-driven approaches. When combined with aroma analysis, flavoromics unlocks new dimensions in the study of food aromas, offering deeper insights into the complex nature of flavor perception based on GC-MS and LC-MS data. Flavoromics integrates chemical aroma data with sensory information to provide a more comprehensive understanding of the flavor contribution of aroma compounds, extending beyond traditional analysis by adopting a holistic approach to understanding flavor perception. Furthermore, flavoromics employs high-throughput data analysis methods, including multivariate statistics and machine learning algorithms, to process the vast amounts of data generated from aroma analysis experiments. These advanced analytical tools can identify patterns and correlations between aroma compounds, food ingredients, processing conditions, and sensory attributes. By applying these methods, Qin et al. conducted a metatranscriptomic study on pickled bamboo shoots and showed that dominant bacterial genera such as *Lactococcus* and *Leuconostoc* regulated the synthesis of organic acids and aroma compounds through the high expression of key enzymes such as lactate dehydrogenase and transaminase. In addition, 13 characteristic volatile components and 68 differential metabolites were identified by combining HS-SPME-GC-MS and UPLC-Q-TOF/MS techniques [123]. Chen et al. also investigated the flavor compound regulation of triploid oysters via 4D-FastDIA proteomics and metabolomics; their results revealed that protein expression was significantly related to umami perception and aroma profiles [124]. Recently, machine learning models trained on aroma analysis data from different batches of a food product were used to predict flavor quality based on the presence and concentration of specific aroma compounds [125].

Flavoromics also enables researchers to investigate the impact of factors such as food origin, processing parameters, and storage conditions on the aroma profile. Moreover, the characteristic aroma marker compounds of food products can be effectively identified based on flavoromics. In summary, the synergy between flavoromics and aroma analysis techniques provides a more comprehensive and in-depth understanding of food aromas. This approach not only reveals the intricate relationships between aroma compounds but also offers practical applications for enhancing food quality and consumer satisfaction. Future research in this field should focus on further integrating cutting-edge aroma analysis technologies with advanced flavoromics methodologies to explore the dynamic and ever-evolving world of food flavors.

## 5. Conclusions and Perspectives

Solvent and solvent-free extraction are the two aroma extraction methods; however, both involve variance in adsorption materials and extraction equipment. SPME and solvent extraction are the most popular aroma extraction methods. However, SAFE extraction is the most popular extraction method for identifying key aroma compounds. Food matrix composition and extraction method selection are two key factors affecting aroma extraction efficiency. The combination of multidimensional chromatography and mass spectrometry with different detectors is an important strategy for the quantitative analysis of aroma compounds, even assisting in problems of co-elution or recognizing unknown compounds detected via traditional one-dimensional chromatography. Since the advent of specific food aroma analysis, characteristic aroma databases have played an important role in decoding food aroma and improving sensory quality, such as the off-odorant database or characteristic odorant database of spicy, meat, plant-based foods, etc. Notably, accurate quantitative analysis is a prerequisite for determining aroma composition and identifying key odorants. Therefore, in the future, using the standard curve and SIDA quantification methods is suggested rather than using half-quantification or even the relative percentage content.

Some challenges and perspectives for future studies include the following: (1) effective adsorbent materials are an important development direction for aroma extraction, which involves increasing adsorption capacity to reduce solvent extraction; (2) automated extraction is an important breakthrough for improving extraction efficiency and reducing system errors; (3) the integration of databases and the use of artificial intelligence in spectral analysis are key to improving aroma identification efficiency and application in food aroma improvement; and (4) the integration of flavoromics enables the decoding of aroma profile contributions and the identification of characteristic marker aroma compounds.

## Figures and Tables

**Table 1:** The 22 keywords with the strongest citation bursts.

No.	Keywords	Year	Strength ^a^	Begin	End	2000–2024
1	Solid-phase microextraction	2000	90.83	2003	2012	▃▃▃▃▃▃▃▃▃▃▃▃▃▃▂▂▂▂▂▂▂▂▂▂▂
2	Potent odorant	2000	43.98	2000	2009	▃▃▃▃▃▃▃▃▃▃▂▂▂▂▂▂▂▂▂▂▂▂▂▂▂
3	Headspace analysis	2000	38.56	2000	2014	▃▃▃▃▃▃▃▃▃▃▃▃▃▃▃▂▂▂▂▂▂▂▂▂▂
4	Impact odorant	2002	34.43	2002	2016	▂▂▃▃▃▃▃▃▃▃▃▃▃▃▃▃▃▂▂▂▂▂▂▂▂
5	Quantitative determination	2000	32.84	2000	2015	▃▃▃▃▃▃▃▃▃▃▃▃▃▃▃▃▂▂▂▂▂▂▂▂▂
6	Red wine	2001	30.2	2007	2016	▂▂▂▂▂▂▂▃▃▃▃▃▃▃▃▃▃▂▂▂▂▂▂▂▂
7	Flavor compound	2000	29.88	2000	2008	▃▃▃▃▃▃▃▃▃▂▂▂▂▂▂▂▂▂▂▂▂▂▂▂▂
8	Character impact odorant	2000	29.46	2000	2009	▃▃▃▃▃▃▃▃▃▃▂▂▂▂▂▂▂▂▂▂▂▂▂▂▂
9	Food analysis	2000	29.29	2000	2005	▃▃▃▃▃▃▂▂▂▂▂▂▂▂▂▂▂▂▂▂▂▂▂▂▂
10	Gas chromatography-olfactometry	2001	27.58	2001	2012	▂▃▃▃▃▃▃▃▃▃▃▃▃▂▂▂▂▂▂▂▂▂▂▂▂
11	Correlation analysis	2020	25.92	2021	2024	▂▂▂▂▂▂▂▂▂▂▂▂▂▂▂▂▂▂▂▂▂▃▃▃▃
12	Aroma extract dilution analysis	2000	24.1	2000	2006	▃▃▃▃▃▃▃▂▂▂▂▂▂▂▂▂▂▂▂▂▂▂▂▂▂
13	Component	2000	24.04	2000	2008	▃▃▃▃▃▃▃▃▃▂▂▂▂▂▂▂▂▂▂▂▂▂▂▂▂
14	Microbial community	2019	21.74	2020	2024	▂▂▂▂▂▂▂▂▂▂▂▂▂▂▂▂▂▂▂▂▃▃▃▃▃
15	Headspace	2001	21.31	2001	2013	▂▃▃▃▃▃▃▃▃▃▃▃▃▃▂▂▂▂▂▂▂▂▂▂▂
16	White wine	2000	21.14	2005	2015	▂▂▂▂▂▃▃▃▃▃▃▃▃▃▃▃▂▂▂▂▂▂▂▂▂
17	Dynamic headspace	2000	20.84	2000	2008	▃▃▃▃▃▃▃▃▃▂▂▂▂▂▂▂▂▂▂▂▂▂▂▂▂
18	Constituent	2000	20.18	2000	2013	▃▃▃▃▃▃▃▃▃▃▃▃▃▃▂▂▂▂▂▂▂▂▂▂▂
19	Cheddar cheese	2000	19.95	2000	2008	▃▃▃▃▃▃▃▃▃▂▂▂▂▂▂▂▂▂▂▂▂▂▂▂▂
20	Volatile thiol	2002	17.62	2008	2017	▂▂▂▂▂▂▂▂▃▃▃▃▃▃▃▃▃▃▂▂▂▂▂▂▂
21	Volatile constituent	2000	15.87	2000	2015	▃▃▃▃▃▃▃▃▃▃▃▃▃▃▃▃▂▂▂▂▂▂▂▂▂
22	Solid phase extraction	2006	15.00	2006	2014	▂▂▂▂▂▂▃▃▃▃▃▃▃▃▃▂▂▂▂▂▂▂▂▂▂

^a^ The strength refers to the sudden increase in frequency or significance of the keyword during a specific time period, typically used to identify hotspots or emerging trends in the research field.

## Data Availability

No new data were created or analyzed in this study. Data sharing is not applicable to this article.
